# Wild Animal Tuberculosis: Stakeholder Value Systems and Management of Disease

**DOI:** 10.3389/fvets.2018.00327

**Published:** 2018-12-21

**Authors:** Eamonn Gormley, Leigh A. L. Corner

**Affiliations:** School of Veterinary Medicine, University College Dublin, Dublin, Ireland

**Keywords:** tuberculosis, *Mycobacterium bovis*, animals, wildlife, stakeholders, value systems

## Abstract

When human health is put at risk from the transmission of animal diseases, the options for intervention often require input from stakeholders whose differing values systems contribute to decisions on disease management. Animal tuberculosis (TB), caused principally by *Mycobacterium bovis* is an archetypical zoonotic pathogen in that it can be transmitted from animals to humans and *vice versa*. Although elimination of zoonotic transmission of TB to humans is frequently promoted as the *raison d'être* for TB management in livestock, in many countries the control strategies are more likely based on minimizing the impact of sustained infection on the agricultural industry. Where wild animals are implicated in the epidemiology of the disease, the options for control and eradication can require involvement of additional stakeholder groups. Conflict can arise when different monetary and/or societal values are assigned to the affected animals. This may impose practical and ethical dilemmas for decision makers where one or more species of wild animal is seen by some stakeholders to have a greater value than the affected livestock. Here we assess the role of stakeholder values in influencing TB eradication strategies in a number of countries including Ireland, the UK, the USA, Spain, France, Australia, New Zealand and South Africa. What it reveals is that the level of stakeholder involvement increases with the complexity of the epidemiology, and that similar groups of stakeholders may agree to a set of control and eradication measures in one region only to disagree with applying the same measures in another. The level of consensus depends on the considerations of the reservoir status of the infected host, the societal values assigned to each species, the type of interventions proposed, ethical issues raised by culling of sentient wild animals, and the economic cost benefit effectiveness of dealing with the problem in one or more species over a long time frame. While there is a societal benefit from controlling TB, the means to achieve this requires identification and long-term engagement with all key stakeholders in order to reach agreement on ethical frameworks that prioritize and justify control options, particularly where culling of wild animals is concerned.

## Introduction

With increased global interest in the emergence of new infectious diseases, the role of animals in the transmission of infection to humans has become a focus of attention (1). The reasons for the spread of infections are complex and multifactorial and can involve changes in human populations and densities, modifications in animal husbandry practices, and changes to the ecological environment leading to human intrusion into wildlife habitats that hitherto remained undisturbed (2, 3). It is the increased risk of transmission to humans that is most often the foundation for efforts to understand the epidemiology of animal disease and the implementation of preventative measures to minimize transmission (4, 5). A case in point is tuberculosis (TB) in animals and the danger it has historically posed to humans. Commonly referred to as “bovine tuberculosis” despite the causative organisms, most frequently *Mycobacterium bovis*, being capable of infecting a wide range of mammalian species, the perceived risk is reflective of the historical close association between livestock and humans (6, 7). During the early part of the twentieth century, in the period preceding the pasteurization of milk, transmission of infection *via* contaminated milk was a serious public health problem in the industrialized world, leading to many thousands of cases of human TB with high mortality rates (8, 9). The discipline of epidemiology (as we understand it today) was then largely non-existent. To the extent that attempts to address the disease in all its forms (cattle and human TB) were driven by competing stakeholder interests (e.g., dairy industry, public health agencies, government), more often than not it resulted in stasis and a complete failure to reduce disease incidence (10). Many countries in Europe eventually achieved eradication of TB from cattle through the roll out of government-regulated compulsory national screening programmes in cattle, and have since maintained this status through monitoring of animals for typical TB lesions at the slaughter house (11). For some of those countries that failed to achieve eradication, despite intensive testing, there was an awareness that the epidemiology of the disease was complicated by other possible sources of infection, notably wildlife (12). This militated against any quick-fix solutions to solve the problems. Instead it lead to decades of research to unravel what has turned out in many circumstances to be extremely complex epidemiology.

There have been few systematic studies worldwide to assess the extent of wildlife TB and it is often the case that studies are only initiated when there is spillover of TB into livestock, or where there is a high value placed on the species by particular stakeholders. Wild animal populations infected with TB are currently found in North and South America, Europe, Africa and Australasia (12). However, the finding of TB in wild animals in any particular environment does not constitute proof that they are a significant source of infection for livestock, companion animals or humans (13). Indeed it reveals little in terms of whether the affected species is a self-sustaining maintenance host or a dead-end spillover host. This distinction is critical for the development of strategies to control the disease in livestock as it can impact on the perspectives and level of engagement among a range of stakeholders. Depending on the reservoir status stakeholders may assign different value systems to the wildlife species and this can directly influence the type of management systems put in place. When TB is found in a free-ranging wildlife population the prevention of spread to other animals, especially livestock is often the immediate priority followed by the prevention of geographic spread. The identification of maintenance hosts is therefore of paramount importance in understanding the epidemiology because the disease can persist indefinitely in the absence of specific management and control programs. If it is established that wild animals are important in the epidemiological cycle and act as a source of risk to livestock, the decision making process as to the preferred actions will primarily depend on the considerations of the reservoir status of the infected host and the broader societal values assigned to each species by stakeholders. With spillover hosts, there may be a broad consensus reached among a limited number of stakeholders that an aggressive response to dealing with the reservoir host is the most effective strategy for stamping out the disease. However, if disease becomes established in a maintenance host, this will attract the attention of a broader range of stakeholders and there will be more complex ethical issues raised from culling of sentient wild animals and the economic cost benefit effectiveness of dealing with the problem in one or more species over a long time frame.

How to deal with the disease problems in cattle, arising from infected wildlife, has in the past often proven to be a quandary for stakeholders, in that government and industry supported measures (e.g., wildlife disease surveillance, culling) were not, at least in the beginning, underpinned by strong scientific evidence (14, 15). Rather, they were often pragmatic choices based on basic, simplistic epidemiological principles that aimed to deliver cost-effective beneficial results to the livestock industry in the short to medium term while awaiting the relatively slow pace of research to decipher the epidemiology and translate the results into policy decisions (14, 16). As a result, the primary driver for disease management in livestock has most often been based on economics and the impact of sustained infection on the agricultural industry (17, 18). In countries where wildlife have been considered as a potential source of infection the programmes evolved as the initial poor epidemiological understanding became clear, both from experience and also resulting from focused research both within the targeted species, and from assessing the risk of spread to other species (16, 19). However, as is often the case with scientific investigations into complex problems there can be an absence of certainty, and this has lead to conflict between the demands of different stakeholders (20).

## Stakeholder Value Systems and Wildlife

“Wildlife stakeholder” broadly describes any person or group with an interest in wildlife. The levels of interest and the weighted values that each stakeholder assigns to particular wildlife can be highly variable, and defining the moral and ethical viewpoints of stakeholders that influences their level of engagement can be difficult. This is because there is likely to be a complex interplay between the values that each stakeholder places on wildlife and how it is linked to their moral perspectives on animal rights, animal-human health, conservation and biodiversity (21). Value systems for wildlife have been broadly classified into a number of groups according to their (a) economic importance, (b) nutritional value, (c) ecological role, and (d) socio-cultural significance (22). Quantifying the values with a high degree of certainty can be problematic as it mostly relies on data collected from surveys assessing preferences of stakeholders (23). Stated preference methodologies, such as choice experiments allow for a structured method of data generation that helps to identify the factors influencing alternative choice scenarios (24). This approach is based on the assumption that individuals will select the choice that they expect will give them the highest benefit (utility), when presented with a set of alternatives. Its advantage over simple stated preference methods is that it allows for the valuation of attributes that characterize a particular scenario, rather than just valuing the scenario itself. Within each scenario there can be a scale of positive and negative values. For example, within the large game reserves in Africa, wildlife conservation activities can have a net positive value because of the significant beneficial impact on the local economy, also through the enhancement of local ecosystems from maintenance of biodiversity, and the cultural significance of wildlife for local communities. Negative values can accrue, for example, if there is crop or other habitat destruction because of over-abundance of particular species (e.g., large herbivores). As another example in the context of TB, choice experiment studies carried out in the UK have shown that badger management policies attract very high values: the surveys revealed that the public places high values on government policies that avoid culling of badgers (25).

Where wildlife start to encroach and compete with human interests negative value perceptions can increase among an expanded range of stakeholders. The divergence of values can lead to conflicts between those who place higher values on human activities (e.g., farming) and livelihoods and those who value the protection and welfare of wild animals. How these differences are reconciled can depend on the environmental and animal ethics perspectives of the stakeholders (26). These perspectives range from a contractarian viewpoint where there is a hypothetical social agreement to manage wildlife wisely for human benefit, to an animal rights focused viewpoint where there is no societal obligation to manage or interfere in any way with the well-being of wildlife. For many stakeholders with a general or transient interest in wildlife the ethical perspectives are likely to represent a blend of different viewpoints combining multiple value systems e.g., utilitarian and animals rights based values, such that respect for wildlife is acknowledged while at the same time adopting a value system allowing for the sacrifice of the interests of some animals for the greater benefit of others. The recognition that wild animals are a source of zoonotic diseases, particularly animal TB, can quickly change the number of stakeholders involved and increase the range of ethical perspectives: it can quickly shift the balance from high values associated with the natural rights of wild animals to much lower values as the threat of TB intensifies. The threat from infected wildlife can, on the one hand, be viewed as a serious agricultural problem with potential significance for broader human activities and health. A contrary perspective can assign higher net values to the affected wildlife species because of the belief that the disease impact is mostly restricted to the livestock industry or that the threat is overstated. Where there is a lack of objective data to support a particular perspective, this can lead to disagreements between those stakeholders who primarily value animal welfare and rights, and those who value the perceived greater benefits to society. Added difficulties arise from trying to define measures of benefit, for example, how can society assess and compare the pain and suffering experienced by slaughter of cattle and culling of wildlife? How are ethical views influenced by the presence of disease in one or both species? Do TB control programmes strike the correct balance between protecting the livestock industry and valuing the benefits of wildlife existing in their natural habitats? If TB was restricted to wildlife, how many stakeholders would be concerned for their fate? From studying the evolution and operation of TB control programmes in different parts of the world, we argue that the presence of TB in wild animals can lead to a change in ethical frameworks, and also involve a wider range and higher level of stakeholder engagement in the strategies employed to deal with the problem. The values of the interested parties appear to be based on an *ad hoc* blend of economic considerations, livelihood activities, knowledge, ethical perspectives, social acceptance, ecological concerns, cultural significance, and political will. This results in significant challenges for the selection of control policies where one or more species of wild animal is seen by some stakeholders to have a greater value than the affected livestock. It can also lead to demands for exceptionally high quality scientific evidence to justify particular interventions. Not all species are of equal significance in the epidemiology of disease, not all are considered equal when subjected to disease management, nor are they always equal in the eyes of stakeholders.

To try and get a better understanding of how policy decisions to manage TB in wildlife are influenced by stakeholders, we have looked at a number of established TB control programmes worldwide where there is strong evidence of epidemiological involvement of wildlife in the transmission of infection. We highlight the influence of stakeholder values on the management of the disease where the contexts differed. The approaches to disease control range from relatively uncomplicated management systems in Australia where there was strong consensus between stakeholders because of the negative value pest status of the wild animals to the highly complex epidemiology of disease in South Africa where multiple species of high positive conservation value are affected and a diverse range of stakeholder groups are involved in the debate on how to control and manage the disease.

## Wildlife TB in Australia

Australia has been uniquely successful in eradication of TB from cattle against the background of a significant wildlife reservoir of infection in an area of one state, the Northern Territory (NT). Eradication was achieved following agreement of key stakeholders to the program, which included addressing the problem of wildlife reservoirs of infection (27). The last known cases of TB in Australia were detected in 2002: two cases in buffalo herds in the NT and a secondary case in a cattle herd in Queensland (28). Studies had revealed that *M. bovis* infection in animals was limited to two maintenance hosts: domestic cattle and feral water buffalo (*Bubalus bubalis*), with infection recorded in only one other wild animal host, feral pigs (*Sus scrofa*) (29, 30). There were only two reports of infection in other domestic grazing animal species: in goats co-grazing with infected cattle (31) and in fallow deer (*Dama dama*) (32). Also, as well as being a maintenance host for TB, feral water buffalo and feral pigs were classified as invasive pest animal species that were causing a major negative impact on the environment of the coastal wetlands of the NT.

The Australian history of bovine TB control mirrors that of other developed countries, with an evolution from a voluntary program in the early twentieth century to a national program commencing in 1970 (33). The initial focus was on removal of diseased dairy cattle to minimize the threat to the human population. Reduction in prevalence was rapid and by the 1960s only a few pockets of infection remained among the southern states where dairy herds were dominant. However, the threat of trade restrictions for meat and dairy products imposed by trading partners in Europe and US lead to the launch of the national Brucellosis and Tuberculosis Eradication Campaign (BTEC), which ran from 1970–1997. The cattle industry was a key stakeholder in this campaign which included herd test and slaughter, compensation payments, tracing of animal movements, all backed up by a dedicated laboratory service. Aerial mustering and ground shooting was used in the large farms in northern Australia with whole herd culling of infected herds during the final stages. It was notable that domesticated water buffalo herds in this region were managed similar to local cattle herds and were subject to a test and slaughter strategy.

Feral water buffalo were only found in the NT having been introduced there in the mid-1800s. In the 1960s the prevalence of TB in slaughtered bulls was 16%. In 1970 at the commencement of the BTEC program, the disease was endemic in buffalo across most of their range (34) with the prevalence of lesions in abattoir slaughtered animals ranging up to 8.2% (35). The buffalo population peaked in the 1980s at around 350,000 head with the majority being unmusterable feral stock. With agreement between some of the stakeholders, that is, state and federal governments, pastoralists and conservationists, a decision was made to eradicate the wild buffalo herds by culling. The culling operations were effective and buffalo were eradicated from the coastal plains of the NT, except for a few domesticated buffalo herds and, at the request of the indigenous Aboriginal land owners, up to 60,000 animals were allowed to remain in the northeast corner of the state, where no TB was ever recorded in cattle or buffalo.

There was strong social, political, and cattle industry support for eradication of feral buffalo with the principal justification being the risk of transmission to cattle, even though there was only limited interaction between buffalo and cattle and no evidence of significant cross-species transmission (13). There was minimal objection to the eradication program from the small commercial buffalo capturing industry. The scientific evidence of damage to the coast flood plains caused by buffalo, leading to saltwater intrusion into the freshwater flood plains, resulting in the loss of habitat for native animals, and birds, was well documented (36). The coastal plains included the Kakadu National Park, a World Heritage site.

When the focus of the Australian national TB eradication program was extended to the pastoral grazing areas of northern Australia there was trepidation among stakeholders as feral pigs were considered as a possible reservoir of *M. bovis* infection. These suids were widespread and numerous in the region, and though the prevalence of confirmed *M. bovis* infection in some studies was high at 19.2% (30) it was subsequently considered from the distribution of TB lesions that they constituted a spillover host with a minimal risk of onwards transmission from pigs to other animal species. It is likely the feral pigs became infected by scavenging on carcases of tuberculous cattle and water buffalo (13). No direct intervention was taken against the feral pig population and it was later shown that after eradication of TB from cattle and the eradication of buffalo, TB prevalence in feral pigs declined significantly (29). Unlike in New Zealand, infection with *M. bovis* was never reported in the common brushtail possum (*Trichosurus vulpecula*). Elsewhere, the absence of infection among native wildlife allowed the focus of the TB campaign to remain on cattle and buffalo. Following the end of BTEC, all subsequent buffalo herds were derived from populations where infection had never been present. Since 2011, infection with *M. bovis* has been classified as an exotic disease of cattle in Australia (37).

## New Zealand and TB in Wildlife

The New Zealand history of bovine TB control parallels that of Australia, starting with voluntary testing of dairy cattle herds in 1941 and moving to stringent and compulsory test and slaughter programmes in 1961 (14, 16). When progress stalled, the discovery of the disease in wildlife was recognized as a possible constraint to eradication (38). Epidemiological studies in New Zealand identified 14 species infected with *M. bovis*, but only three, domestic cattle, domestic deer and brushtail possums, were identified as maintenance hosts, though wild ferrets (*Mustela furo*) were considered as possibly a maintenance host in very limited areas (16, 39). Although not considered as maintenance hosts, feral pigs and wild deer, along with the ferret, have proved invaluable as sentinel hosts for surveillance of TB in possum populations (40). The current testing program for cattle and deer is based on the risks associated with transmission of infection from possums (14). The brushtail possum is a small arboreal marsupial, first introduced into New Zealand from Australia in 1837 to establish a commercial fur trade (41). They were officially classified as a pest species in 1948. The possum population reached a peak of around 50–70 million in the 1980s. The original public perception of possums as harmless changed when it was shown that they might pose a great threat to survival of native fauna, including the iconic kiwi (42). Although first shown to be infected in the mid−1960s, the findings in the 1970s revealed that possums were a maintenance reservoir host for *M. bovis*, and strongly implicated in the transmission of infection to cattle. Studies also showed that possums were highly susceptible to infection resulting in a rapidly disseminated and fatal disease (41, 43). Although generally avoiding cattle, terminally ill possums display abnormal behavior patterns which could bring them into contact with inquisitive cattle (44, 45).

The early government-led initiatives to control TB in cattle subsequently evolved into a public-private partnership between the government and the livestock industries with a remit to conduct wide scale possum control (16). The objective of the national program was to eradicate *M. bovis* from New Zealand and this received general societal and industry stakeholder support (46, 47). The broad geographic distribution of tuberculous possum populations and the large number of other species affected initially made the prospects of eradication unlikely even though there was support for the TB eradication program, especially the focus on possum culling (48). In recent years a choice experiment survey of the NZ public was carried out to assess the non-monetary benefits to native forest biodiversity arising from TB-related possum control (48). This revealed strong public stakeholder support for the benefits of possum control, particularly the values placed on the observable effects of improved forest canopies and the positive impact on native bird, insect and plant species. The main criticism of possum control has subsequently been aimed at the methods used to cull possums, especially the use of sodium fluoroacetate (1080) by aerial application (49, 50).

The early possum control measures helped to significantly lower the incidence of disease in cattle herds, but relied on basic assumptions of the epidemiology, rather than any hard scientific evidence (41, 51). Where large scale possum control measures were successful, the TB levels in the sentinel species also declined, demonstrating that targeting resources at one key maintenance reservoir had a direct beneficial effect on other species (52). Currently the population of possums is estimated to be in the order of 30 million. As a result of the possum culling, also controls on the movement of cattle and deer, and TB testing, the number of infected herds in NZ has dropped from ~1,700 deer and cattle herds at the peak in 1995 to 41 herds in 2015 (14, 16).

## Badger TB in Ireland and the UK

In recent times the most controversial wild animal TB control strategies in Ireland and the UK have revolved around the European badger (*Meles meles*) with deep polarization of opinion among many of the stakeholders, particularly in the UK (53). The role of badgers in the epidemiology of TB in cattle in the UK and Ireland has been subject to intensive investigations since *M. bovis* infection was first identified in badgers in England in 1971 and subsequently in Ireland in 1974 (54, 55). Over the preceding 10 years substantial progress had been made in reducing the incidence of TB in cattle in both countries due to mandatory herd screening programmes (9, 56, 57). When progress stalled, and badgers were found to be infected, local badger culling operations resulted in an apparent decline of disease in cattle (57, 58). Over the next two decades evidence accumulated through large scale culling studies that strongly implicated badgers in the TB transmission cycle (59–63). The advent of DNA genotyping of *M. bovis* isolates also revealed that prevalent genotypes were common in both cattle and badgers sharing the same environment, providing evidence of cross-species transmission (64, 65). Tuberculosis in badgers is a chronic slowly progressive disease (66) and infected badgers satisfy the criteria to be a maintenance reservoir host for *M. bovis* in Ireland and the UK (13). They are highly susceptible to infection and the predominant location of lesions suggest that infection among badgers occurs principally *via* the respiratory route with transmission from infected bite wounds being of secondary importance (67, 68). The social structure of badgers facilitates close interactions that lead to an increased risk of transmission. Pseudovertical transmission from dam to cub is likely to be a key factor in maintenance of infection within local populations (66).

In the Republic of Ireland the national TB eradication plan commenced in the late 1950's, and the strategy has succeeded in decreasing TB incidence in cattle and maintaining it at a relatively low level (69). This has been achieved using a program of sustained cattle testing and targeted badger culling (70). Prior to the implementation of a national badger culling strategy in the Republic of Ireland, two separate badger culling studies (East Offaly Project and the Four Area Study) confirmed the role of badgers in the epidemiology of TB in cattle. Both trials showed a significant drop in cattle TB prevalence in areas where badgers were proactively culled in comparison to the control areas (60, 63). A separate study conducted in County Laois between 1989 and 2005 also provided evidence that badger culling had a positive impact on the risk of future TB breakdowns in cattle and a positive protective effect on herds neighboring the index herd (71). Badger culling was incorporated into the national TB eradication strategy in 2004. As a compromise with stakeholders who had reservations about the strategy, there was a limit imposed on which individual setts could be culled in the relation to the index herd and the proportion of the badger population subjected to culling. Since then, the Irish culling program has focused on areas with high incidence of infection in cattle; areas in which studies have shown the highest infection prevalence in badgers (72). Culling is only conducted following an exhaustive epidemiological investigation to rule out other causes of herd breakdowns (e.g., residual infection, contiguous herd spread, purchase of undiagnosed infected animals), and where badgers are considered as a likely source of infection. Analysis of data generated from culling studies has shown a beneficial long-term decrease in cattle TB (71) and also TB in the badgers of the re-emergent population (73). The culling of badgers in Ireland at national level is considered as an interim strategy to minimize transmission to cattle pending the development of a suitable and effective vaccine. Most stakeholders have accepted culling of badgers, albeit with reservations (72). These reservations are mainly framed around the evidence base that implicates badgers in the epidemiology of TB in cattle, that there is an effective control programme in place for infected herds, and whether culling of badgers is an acceptable measure when the benefits to cattle are difficult to quantify against a background of other control measures focused on cattle.

In the UK there are a large number of stakeholders with diametrically opposing views involved in the debates on the TB control strategy. Culling of badgers to control TB in cattle has proved extremely controversial since it commenced in 1973. Concerns over badger welfare arose from the Ministry of Agriculture, Fisheries and Food (MAFF) policy of gassing setts with hydrogen cyanide, leading to a number of commissioned reports over the following decades, with no clear resolution as to how the scientific evidence should inform policy. The Zuckermann review in 1980 recommended sampling of badgers in the vicinity of affected farms and culling at setts if badgers tested positive (74). In 1986 the Dunnet report questioned the efficacy and the cost sustainability of this “clean ring” strategy (75). The Krebs report concluded that though the evidence was indirect, badgers were a significant source of infection in cattle and recommended an experimental trial to quantify the impact of badger culling on cattle TB (76). The Randomized Badger Culling Trial (RBCT) was carried out between 1998–2006 with the objectives to generate scientific evidence for the role of badgers in cattle TB, and to help formulate appropriate policy measures. However, it ended up highly divisive and the legacy of the trial continues today. Cassidy (77) argues that the design, scale and complexity of the trial, including ongoing disruption by anti-cull protesters made it extremely difficult to generate a strong evidence base that might have otherwise been gathered in more conventional small case controlled studies. The trial area consisted of 10 sets of “triplets” areas, each containing a proactive-culling area, a reactive-culling area with culling only in response to a cattle TB cattle breakdown and a survey-only area where no culling was conducted. The trial results showed that the incidence of bovine TB in cattle dropped by 19% in the proactive-culling area. However, the proactive culling was also associated with a 29% increase in cattle TB in the area outside the culling zone (62, 78). The increase in cattle TB outside of the culling area was attributed to the “perturbation” effect, where the social behavior of badgers was altered by the culling activities, leading to increased interactions and transmission rates to cattle in the area outside of the badgers normal territories (78, 79). Reactive culling was abandoned early in the trial as it was believed to increase, rather than decrease, the incidence of TB in cattle in these areas.

Since its completion, the conclusions of the RBCT have been disputed and the data re-interpreted many times. In its final report, the government appointed Independent Science Group (ISG), which oversaw the trial, concluded that “*badger culling can make no meaningful contribution to cattle TB control in Britain*” (78). This viewpoint was somewhat contradicted by the (also-) government commissioned follow-up King review which found that badger culling “*could make a significant contribution to the control of cattle TB in those areas of England where there is a high and persistent incidence of TB in cattle, provided removal takes places alongside an effective programme of cattle controls*” (80). Cassidy (77) points out that the ISG took a broad perspective to their remit and combined analysis and policy issues to reach their conclusions, whereas the King review restricted the focus to the scientific evidence, without taking account of policy considerations and animal welfare concerns. The situation has not been helped by the perceived inability of successive governments to formulate a long-term policy that balances the pros and cons of the moral arguments used by stakeholders. Changes in the UK government over the years has lead to major policy shifts on badger control measures, further emphasizing the inability of government stakeholders to implement evidence based policies while taking account of the viewpoints of interested parties (53, 77). The Labor government of 2006–2010 accepted the findings of the ISG and resisted pressure from the Nation Farmers Union (NFU) and the British Veterinary Association (BVA) to endorse culling. The Conservative—Liberal Democrats coalition government (2010–2015) changed policy and agreed to introduce culling on a limited scale. While the majority of politicians and many stakeholder groups recognized the role that badgers played in the epidemiology of TB in cattle, there was less agreement on the strength of the RBCT scientific evidence and also the economics and ethics of large scale culling. Pilot culls commenced in Somerset and Gloucestershire in 2013 attracting major opposition from a large cross section of community groups and organizations. When it was reported that the trials failed to meet the pre-determined limits of humaneness and efficacy, this served only to galvanize opposition that demanded a cessation of culling. The effectiveness of the culling operations was also questioned and despite all of the confounders associated with the design (e.g., failure to achieve reduction of targeted population, differences in levels of implementation), it appeared that badger culling was associated with a reduction in cattle TB incidence (81). As cattle TB rates continued to climb in the UK, the Conservative government in 2015, although fully attuned to the unpopularity of culling, expanded the culling areas to placate the demands of the farming industry. Elsewhere, contrasting policies operated in other parts of the UK experiencing problems with cattle TB. The Assembly in Wales has resisted a badger culling policy but instead has increased the range of cattle control measures and focused on vaccination of badgers (82). In Northern Ireland the local Assembly agreed to a badger Test, Vaccinate and Release (TVR) trial to gauge the effectiveness of this approach on cattle TB rates. The strategy involves capturing live badgers in an area with a high level of cattle TB, testing the badgers for TB, vaccinating those that test negative to the disease and removing those that test positive (83). A survey of farmers attitudes to TB control strategies in Northern Ireland revealed a willingness to allow vaccination and culling of badgers on their land with an overall preference for vaccination, and less concern about public opposition (84).

The multi-dimensional aspects and complexity of the evolution of cattle TB policy in the UK raises many questions on the ethics and value systems of stakeholders in the context of culling of badgers. The role of the media is of key importance in framing the viewpoints of many of the principal actors (85). Where there is difficulty in understanding the complexity of the scientific evidence, the press can influence perspectives by over-simplifying the arguments for or against a particular strategy e.g., culling or vaccination, and this can help to fuel the controversies. This can lead to poorly thought out policy decisions, which may reinforce perceptions of mismanagement. Surveys of farmers in new endemic TB areas in the UK have revealed a fatalistic attitude to the problem, where many believed that bad luck played a role in herds contracting TB, but also mediated by perceptions of the political aspects of the disease and the lack of trust in government (86, 87). Similar perceptions were found in Northern Ireland where interviews with focus groups (cattle and beef farmers, private and state veterinarians) revealed differences in perceptions and knowledge of the disease among the different actors (88). It was concluded that a “one-size-fits-all” approach to control policy would be unlikely to succeed without recognizing the heterogeneities of many aspects of disease transmission and the multiple framings of the disease by different stakeholders.

McCulloch and Reiss have described the history and evolution of government policy toward control of TB in badgers in the UK (53). They argue that the debate can be distilled into two questions related to quantifying the role that badgers play in the epidemiology and whether the current control options are effective, practical (in controlling transmission) and socially acceptable? They conclude that policy should not be based exclusively on scientific evidence, economics or public opinion. Rather, they propose that the ethical issues need to be addressed by independent experts according to moral frameworks that question what is right, and what is justifiable, and taking into account the impact of policy on the morally affected stakeholders. McCulloch and Reiss separately analyse these frameworks from a utilitarian perspective (89). This approach strives to achieve a balance between the competing interests of stakeholders in order to achieve the greatest utility benefit for all. But it raises the question as to who are the greatest beneficiaries and how can one measure and quantify the utility benefit? In this context there is a generally perceived human benefit from farming arising mainly from production of high quality food leading to good public health. But there is also a strong societal benefit and positive value from maintaining undisturbed badger populations in their native habitats (25). Because of TB there are conflicting viewpoints on these utility benefits among stakeholders. McCulloch and Reiss argue that according to utilitarian theory, “*the slaughter of a cow or the culling of a badger with a life of net positive value will result in a loss of utility. All else being equal, the killing of a cow or badger that could be expected to continue with a life of net positive value is, therefore*, prima facie *morally wrong, simply because it reduces total utility*” They suggest that killing of badgers can be morally justifiable if it results in greater overall utility, e.g., the replacement of the (badger) utility by cattle, or an increase in human utility through improved farming economic benefits accruing from culling of badgers. They then pose the question as to how much culling of badgers is acceptable to justify the increased utility value of cattle? The contention from the ethics of utilitarianism is that the correct policy is one which produces overall highest utility. But this relies on an understanding of the consequences of the policy such that the impact of different policies can be objectively compared and measured. Their analysis concluded that non-culling approaches including badger vaccination policy options resulted in higher overall utility, and was superior to the badger culling option. In the absence of agreement among stakeholders, vaccination of badgers offers a utilitarian solution, and is now considered as a strategy that can address many of the negative issues associated with culling (90).

## Wildlife TB in Spain and France

In continental western Europe, Spain is considered to have a complex epidemiology of TB involving multiple mycobacterium species, animal species and several maintenance hosts including cattle, deer and wild boar (91). Domestic goats, sheep and free-ranging domestic pigs are also implicated in the transmission cycle and often share common pasture land with cattle (92–94). Infections with *Mycobacterium caprae* is common in goats and has been known to spill over to cattle (92). Badgers have also been shown to be infected with *M. bovis* though the impact on livestock is unclear and may differ according to the region (95). For example, badger numbers are more abundant in the cooler Atlantic influenced regions in the north of the country where molecular typing of *M. bovis* strains has shown that they are common to badgers and cattle (96). In the southern mediterranean region of Spain wild boar are believed to be maintenance reservoirs of infection (97). These animals are well-adapted to the seasonal variability in food and water sources, and their mobility and scavenging on infected carrion (e.g., deer) likely influences the pathogenesis of disease which is frequently associated with head, pulmonary and disseminated TB lesions (97). Wild boar are considered as a significant risk factor for TB breakdowns in cattle (98), likely resulting from indirect contact (99).

High densities of wild boar are often maintained by artificial feeding to support a vibrant hunting industry, typically in fenced game estates many of which also house deer, cattle, sheep and pigs in free ranging systems (100). During the hot season experienced in southern regions of Spain, wild boar and other wild species congregate at high densities at watering holes increasing contact rates and the probability of both transmission within and across species (101). Surveys of *M. bovis* prevalence in Doñana National Park (DNP) in southern Spain have revealed prevalences of 52% in wild boar, 27% in red deer and 18.0 % in fallow deer (102). In areas where cattle are absent, prevalences have reached 92% in wild boar (102). The congregation of boar at feeding sites is associated with the high risk of tuberculosis in deer (103). The DNP is also one of the last refuges of the critically endangered Iberian lynx (*Lynx pardinus*), which along with foxes (*Vulpes vulpes*) are considered as spill over hosts (104, 105). In comparison, the prevalence of TB in wild boar in the Atlantic influenced habitats of Northern Spain is significantly lower when compared with the mediterranean habitats (96). This is likely due to lower densities in the northern regions, lower levels of artificial management, less congregation at water holes; all of these factors impacting on infection transmission rates.

As in many other European countries TB eradication in Spain was initially driven by the high prevalence of disease in cattle. When research revealed a multi-host epidemiology of disease, this brought additional stakeholders, including government, hunting lobbies, agricultural industry, and conservationists into the discussion on how best to manage the problem. Culling of wild boar has been shown to be an effective and strategic measure to reduce prevalence, and with the added likely benefit of a decrease in transmission to other species (106). However, culling of animals has also caused conflict among stakeholders while policy makers have attempted to balance the competing interests of hunters, producers, and conservationists. The principal reason is because the hunting estates require managed high densities of animals to maximize commercial returns and hunting groups are resistant to widescale culling (107).

Research has continued in Spain to monitor changes in the occurrence of TB and to unravel the complexities of the epidemiology with a long term view to measure the impact of interventions that may reduce transmission rates among all affected species (108). A questionnaire survey was carried out among key stakeholders (veterinarians, livestock owners and farmers) in south central Spain to gauge their opinions on specific intervention strategies chosen by a panel of experts that included veterinarians engaged in research into wildlife and disease management in the study area (20). Although banning of supplementary feeding of wildlife on cattle farms was ranked by the experts as the most effective control option, this opinion was not shared by hunters and farmers as a practical measure. Overall, hunters and farmers showed the highest levels of agreement for the top-ranked interventions (ban on supplementary feeding, restricting access to waterholes, increased frequency of cattle testing, removal of discarded offal from hunting land) while hunters and veterinarians agreed least. This study highlighted the diverse attitudes of different stakeholders to a range of intervention strategies and probably reflected differences in opinion on the broader epidemiological picture. The opinions of farmers and hunters were more aligned because of their converged interests in the same parcels of land required for their activities, whereas the perspectives of veterinarians were primarily guided by principles of disease management while trying to balance the interests of all stakeholders including policy makers (20).

France was declared officially TB free in 2000, but since then sporadic outbreaks of TB in cattle have continued in a number of regions in the south-west and east of the country (109, 110). TB was also first identified in wild red deer in the northern Normandy region of France in 2001. By 2006, prevalence rates remained high in deer (24%) and wild boar (42%) despite culling of these animals to reduce densities (111). TB infection was subsequently diagnosed in badgers in the areas most affected by TB in cattle (112). Arising from increased concerns over broader wildlife involvement in cattle TB outbreaks (TB in wildlife occuring in areas with cattle TB), the French General Directorate for Food (DGAL) and institutions involved in animal health and wildlife management established a national surveillance system “Sylvatub” in 2011 (113). This serves to co-ordinate the activities to detect and monitor TB in wildlife, and involves a wide range of national and local stakeholders including hunting and wildlife agencies, cattle breeders, pest controllers, trapper associations, veterinary associations and public administrators. The objective is to develop a broad national understanding of the risks associated with TB in wildlife allowing for the design and implementation of control strategies. An evaluation of stakeholder perceptions of the Sylvatub has revealed overall satisfaction with the system, the utility of helping farmers being the primary motivating factor (114). The improved understanding of TB epidemiology was also cited as a motivating factor for participation. Disincentives to participation included practical difficulties, regulatory hurdles, time-consuming activities, economic and material constraints. The results of this evaluation appear to feed into the same stakeholder narrative in other countries experiencing wildlife TB problems, in spite of a low impact on TB rates in cattle.

## TB in Wildlife in USA

The success of TB control in a wildlife species can crucially depend on the support or otherwise of individual or groups of stakeholders. In the USA white-tailed deer (*Odocoileus virginianus*) are the principal wildlife maintenance hosts implicated in transmission of TB to livestock in Michigan and Minnesota (115). Although there have been significant differences between the two states in the prevalence of TB in deer and the size of areas containing infected deer, the responses to the disease have been contrasted by temporal, social, economic, and logistical factors.

The US National Bovine TB Eradication Program was launched in 1917 following years of often fractious debate on the merits of different control options based around meat inspection and the recently developed tuberculin skin test (8, 15). Pasteurization of milk for human consumption had commenced almost a decade earlier in Chicago and New York, and other major cities, to reduce the risks associated with zoonotic TB and other diseases. Stringent application of the test and slaughter control measures lead to rapid success in controlling the disease and by 1940 prevalence was reduced to < 0.5% in every state (116). Prevalence in livestock was recorded as 0.003% in 1994. Between 2001 and 2011, 92 US cattle herds were diagnosed as TB infected and several constraints to eradication were identified including changed management practices, importation of infected animals and the emergence of the disease in cervid species, particularly wild white-tailed deer in Michigan and Minnesota (116).

The disease was first reported in wild deer in Michigan in 1975 and was considered an isolated event (117). In 1994 it was detected again in a hunter shot deer close to the original case, providing for early but inconclusive evidence of a possible wildlife reservoir. It was the impetus for the state to initiate a TB control programme targeted at wildlife and farmed deer. In 1995 after the first year of systematic wildlife surveillance, 4.9% of deer sampled were culture positive for *M. bovis* in the core outbreak area (~1,500 km^2^) (118). With the disease eradication plan underway, addressing both the deer and the cattle populations, there was resistance mounted by some of the large number of stakeholders, with no universal acceptance for the proposed control measures (119). While there was overall support among deer hunters, livestock producers and agricultural business for the eradication of TB, there were differences in the knowledge and perceptions of the threats of TB, leading to a lack of support for eradication measures (120). As a major stakeholder, the hunting industry did not consider that the extent of the disease problem warranted reduction of deer numbers in the infected areas, and they were opposed to the banning of supplemental feeding and baiting which had helped to increase deer densities. From an epidemiological perspective, this provided opportunities for contact between infected and susceptible animals either by direct contact or contamination of food (121, 122). Agricultural producers relying on crop production for sale of feed to the hunters also considered the measures as a threat to their business. Among livestock producers, including those with most to lose from the TB outbreaks, only 57% supported further reductions in deer numbers. These differences in values among the key stakeholders and problems with compliance constrained the ongoing control efforts and TB in cattle and wildlife (123, 124) and TB breakdowns in cattle continued, preventing the state from regaining its former TB free status. Between 1994 and 2010 there were however only 50 cattle farms positive for TB, and of those within the TB core area the most likely source of infection for the herd was wildlife. The majority of TB infections in other wildlife including coyotes (*Canis latrans*), racoons (*Procyon lotor*), red fox (*Vulpes vulpes*), bobcat (*Felis rufus*) and black bear (*Ursus americanus*) have been found in the northern portion of the Michigan's Lower Peninsula which contains the core area and probably amounts to them being spillover hosts (125). The full state of Michigan has still not regained its TB free status from the USDA (123).

When TB was detected in white-tailed deer and cattle in Minnesota in 2005 there was a rapid and aggressive response (119). The control of TB was framed by implementation of a strong management programme by the Minnesota Department of Natural Resources. The outbreak was confined to a small area of < 425 km^2^. By 2011 only 12 beef cattle herds and 27 white-tailed deer had been diagnosed with TB. The result of studies to investigate the factors associated with deer-cattle transmission had implicated deer visits and damage to stored cattle feed as a major risk factor (123). The decision was made to eradicate infection from both the cattle and deer populations by culling both species and this inevitably placed an economic burden on both the cattle industry and recreational deer hunters. A new deer management unit was created that allowed for additional hunting opportunities. Private landowners were issued with shooting permits to remove an unlimited number of deer on their lands, with the proviso that samples were submitted for TB-testing and the carcasses used for venison, thus avoiding wastage. A recreational feeding ban for deer and elk was put in place in 2006 in the TB endemic areas and monitored by enforcement officers. The plan was highly successful in reducing the prevalence of TB and by 2011, there were no recorded cases of TB in deer or cattle in Minnesota (119). As a result the state re-gained its TB free status from the USDA (119).

Although the key stakeholder groups in Michigan and Minnesota were similar and likely motivated by the same concerns, the outcomes of the TB eradication programmes in each state were markedly different. There are a number of factors that may have contributed to the divergent outcomes. The control efforts in Minnesota benefited from the issues revealed from the interventions of the Michigan campaign. With the disease emerging much later in Minnesota there was political pressure to quickly stamp out the disease before it became endemic. Thus, control measures were implemented much earlier after discovery of the outbreak in Minnesota, whereas the disease was present for at least 20 years before control measures were applied in Michigan. Although there was some resistance to deer culling from hunters in Minnesota there was also the realization that TB eradication in the short term was beneficial to the industry in the long term. The demands for strong action from the cattle industry also made it easier for politicians to implement aggressive actions.

Carstensen et al. describe a combination factors that may have contributed to the different levels of stakeholder acceptance in both states and the more aggressive response in Minnesota (119). They highlight that the core area of the TB outbreak in Minnesota was 29% of that in Michigan. Also, the terrain topography and the substantially higher proportion of publicly owned land in Minnesota facilitated access for shooting of deer. Use of helicopters for aerial shooting to remove deer was controversial, though strong engagement with all stakeholders through public meetings helped to alleviate concerns. While baiting and feeding of deer were illegal in the core outbreak areas of both Michigan and Minnesota, baiting was illegal in Minnesota more than a decade prior to the finding of TB in cattle or free-ranging deer. The land ownership in Michigan's core area comprised 90% private land, including hunting areas, making it difficult to enforce compliance with the law. The number of farms in the affected area of Minnesota was twice that of Michigan's core area, helping to increase the political clout of the cattle industry in Minnesota. A buy out program was available to cattle producers in Minnesota's TB outbreak area to help reduce the cattle population at risk. A high proportion of eligible farms accepted the buy-out, and ~6,200 cattle were removed from the TB affected area. A similar buy out was not facilitated in Michigan. What these factors illustrate is how differences in the value systems of the same stakeholders in each state affected the outcome of the disease eradication measures. From a value systems perspective the utility value of the deer in Michigan was given a higher overall nominal score because of the powerful hunting lobby, whereas, in Minnesota the concerns of the agricultural lobby trumped the hunting industry allowing the state officials to implement a much more forceful control plan.

## Wildlife TB in Africa

The number of wild animal species involved in the highly complex epidemiology of TB in South Africa poses particular challenges for identifying and engaging with stakeholders in order to seek broad consensus on control strategies (126). The African continent is home to a vast and diverse range of indigenous wild mammals, many, if not most, of which it can be assumed are susceptible to infection with TB (127). Given the lack of any reliable hard data, it is not known with certainty if the disease was originally introduced by human activities or if it always had a presence in wildlife at some level, with the open and expansive landscape facilitating interactions and new incidents of infection across multiple species (128). The advent of molecular typing of strains isolated from cattle has revealed the presence of three geographically distinct *M. bovis* clonal complexes in Africa, the African Af1 complex dominant in sub-Saharan West-central Africa (Mali, Cameroon, Chad and Nigeria), African Af2 found in East Africa (Uganda, Burundi, Tanzania and Ethiopia) and European Eu1 complex in South Africa (129–131). The presence of the Eu1 strain is associated with the arrival of the Dutch and British colonial settlers in South Africa with TB infected cattle, and represented a significant event in the emergence and spread of TB among native animals.

TB was first identified in cattle in South Africa in the late nineteenth century, and in indigenous kudu (*Tragelaphus strepsiceros*) in 1928 (127). In the following decades the disease was diagnosed in an increasing number of wildlife species including duiker (*Sylvicapra grimmia*), and springbok (*Antidorcas marsupilias*). More recently research has focused on the Hluhluwe-iMfolozi Park (960 km^2^) and Kruger National Park (19,485 km^2^) where it is believed that TB was transmitted to the African buffalo (*Syncerus caffer*) from domestic cattle in the 1950s (132). Among the many wildlife species affected the buffalo is considered to be the principal maintenance reservoir of infection, although kudu also appear to maintain the infection (132–134). By 1995, the disease had spread northwards from the southern part of the Kruger and since then has affected many different animal species including lion (*Panthera leo*), cheetah (*Acinonyx jubatus*), kudu, leopard (*Panthera pardus*), chacma baboon (*Papio ursinus*) (135–137), black rhinoceros (*Diceros bicornis*) (138, 139) and white rhinoceros (*Ceratotherium simum*) (140). There was also evidence of spillover to neighboring livestock (141). Molecular strain typing has shown that the infection had spread by clonal expansion of the Eu1 strain type and spread to game farms and reserves in Mpumalanga, Limpopo, KwaZulu–Natal, Free State and North West Provinces, affecting at least 16 different animal species (142).

In South Africa a voluntary test and slaughter scheme for cattle was initiated in 1969, and by 1991 had reduced the disease prevalence to 0.04%. However, primarily due to financial and resource reasons this level of success was not sustained, and the disease levels increased thereafter (132).

There is only limited basic epidemiology known for most African wild mammal species other than buffalo (143). As the disease became established in maintenance hosts it was inevitable that the infection transmitted to predator species, including lions, hyena (*Crocuta crocuta*), leopard and cheetah, and a range of scavengers and omnivores (142). These, as with other predators, are probably spillover hosts where the infection is unlikely to be sustained in the absence of external sources of infection. The pattern of generalized TB in the prey species (including buffalo and antelope species) increases the likelihood of transmission following ingestion of infected organs and tissues.

In South Africa, all aspects of wildlife have provided lucrative business opportunities with increased global interest in ecotourism, trade in wild animals and conservation (132). The number of wildlife has increased considerably in South Africa in recent years, both in national parks and private game reserves. Iconic African wildlife species are exported worldwide to zoos for conservation and can attract very high purchase fees (139). In the absence of any reliable ante-mortem diagnostic tests for TB this poses great challenges to controlling spread of infection when animals are translocated to reserves within Africa or exported worldwide. There are many recorded examples of tuberculosis in rhinoceros housed in zoos going back over 100 years yet in that time there have been relatively few advances in development of sensitive diagnostic tests other than relying on observation and clinical symptoms (139). The finding in the Kruger National Park of an infected free-ranging black rhinoceros (138) and in the white rhinoceros (140), species recognized as critically endangered and near threatened by the International Union for Conservation of Nature, has serious implications for the conservation measures for rhinoceroses, and movement out of the Park for breeding and conservation reasons.

With the expanding range of African animals infected with TB, it is difficult for programme managers to deal with the problem given the enormous costs involved, notwithstanding the paucity of epidemiological information available for single species let alone unraveling the complexities of the infection in multi-species hosts (132, 144). The deficiency in the epidemiology of the multi-host system prevents any single proposed programme from claiming precedence. In South Africa control of animal diseases is regulated by the Department of Agriculture, Forestry and Fisheries (DAFF) though there are many local, national and international stakeholders involved, including ecologists, veterinarians, conservationists, animal rehabilitation centers, ecotourism companies, game capture operations, national and provincial parks, hunting companies, the cattle industry, wildlife ranching etc. Given the diverse range of the interest groups, there is likely to be as many conflicting opinions on how to manage the problems. For example, although TB is endemic in many buffalo populations, it does not appear to be detrimental to their population structure, nor are TB test positive buffalo more likely to be subjected to predation by lions (145). This may lead to opposing viewpoints from those groups who believe the presence of TB has minimal ecological impact and, for example, veterinarians motivated to eradicate disease. Spillover of disease to high value predators does raise concerns from many additional stakeholders. It is unlikely that TB can now be eradicated from the community of affected species by current available methods and policies are likely to be framed around management of the disease to minimize spread. Test and slaughter programmes, if available, may serve to decrease local prevalence but are unlikely to achieve eradication. Resources may be focused on species of highest monetary or conservation value, thus providing short to mid range economic benefit but achieving little in the context of eradication of the disease from free-living animals. Vaccination may provide a potential solution in the future, however it would need to be cost-effective, and any chance of success will also require many additional studies to improve epidemiological knowledge and understand how control measures directed at one or more species affects the dynamics of disease in multi-host systems (146).

## Vaccination of Wildlife Against TB

Where culling of animals is not considered a feasible option (for whatever reason) as a disease management tool, vaccination of wild animals against TB is often promoted as an alternative strategy, primarily because it provides for a non-destructive approach to controlling disease and addresses animal welfare concerns, as well as conservation concerns arising from deliberate killing of wild animals (147, 148). The purpose of vaccination is to reduce the incidence of infection leading to lower levels of intra-species spread of infection, as well as transmission to other wild species and livestock (149). By reaching and maintaining a threshold level of coverage the vaccine will also confer protection to the non-vaccinated proportion of the population through the generation of herd immunity. Over time, and with an effective vaccine, the disease will eventually disappear from the vaccinated population. The BCG vaccine, used extensively in humans, has been shown to work in a variety of animal species (147, 150, 151), and more recently an alternative heat inactivated *M. bovis* (HIMB) vaccine candidate has shown some promise in a range of species (152–154). These vaccines can be delivered by injection or oral bait. BCG is a live vaccine and a single dose can provide a long duration of protection against natural exposure to infection (155).

In deciding on the appropriate control strategies to employ, the desired outcome needs to be carefully considered in order to avoid further conflict among stakeholders. For example, vaccination of badgers may address conservation concerns arising from culling a protected species, with the added benefit of protecting cattle from badger—cattle transmission. However, the time frames to achieve eradication will be much longer when compared with culling (156). Studies of UK farmers' perceptions of vaccination as a means to control TB have also revealed cautious attitudes to this strategy (157). It has been noted that the media paid more attention to vaccination when the controversies over culling escalated (85), and wildlife groups have heavily promoted the vaccine strategy. While there is good field data to show that the vaccine can protect badgers in their natural environment, the scientific evidence of a direct link between badger vaccination and time scales for a positive impact in reducing TB breakdowns in cattle is lacking. This serves to reduce farmers' confidence in vaccination, which in part reflects their lack of trust in the ability of government to control the disease (86). There is also a viewpoint among farmers of over-population of badgers that is consistent with a preference for culling of badgers above vaccination (158). If farmers believe that there is little that can be done to control the disease, a vaccination strategy is also unlikely to alleviate such concerns. Elsewhere, BCG vaccination may be of use in countries without established control or eradication programmes where testing and slaughter of reactor cattle is not practiced or considered acceptable for economic, social or religious reasons.

## Discussion

The eradication of TB from animals has faced many challenges since studies commenced in cattle, when in the early 1890s Koch's old tuberculin was found to be useful as a diagnostic tool for TB (159). Along with pasteurization of milk and slaughter of infected animals, these measures would eventually herald a new age where the impact of zoonotic TB was effectively controlled. From today's perspective it seems extraordinary to consider that stakeholders did not universally welcome these approaches as a potential panacea to reduce the burden of infection in humans. To understand this we must take account of some of the value systems that underpinned opposition to the policy at the time. In the US, which launched its TB eradication program in 1917, when TB was causing greater morbidity and mortality among cattle than all other diseases, there was often complacency and resistance to mass tuberculin testing of animals (8, 15). The TB problem was seen as wholly intractable and any broad scale measures would result in unacceptable economic losses. At that time there was also considerable resistance, particularly in the UK, from the dairy industry to any government imposed interventions that would increase production costs and where the benefits were largely unproven (10). During this period it was primarily veterinarians who supported the campaign of compulsory inspection, animal slaughter, pasteurization, and any other measures that might help to eradicate the disease (10). However, there were also many in the profession whose livelihoods depended on the custom of farmers and were opposed to some of the proposed measures. Given the high burden of disease in cattle there was also the view among interested parties that mass screening and slaughter of infected animals would decimate the dairy industry (10). The historical record highlights the different perspectives of stakeholders in dealing with a serious zoonotic disease, which in the end only succeeded in stalling progress to reduce the incidence of zoonotic TB. The emergence of the discipline of epidemiology in the past fifty years has increased our understanding of many of the risk factors associated with TB in cattle and wildlife, but it has also generated and molded the viewpoints of many different stakeholders. There is now better knowledge of wildlife sources of TB infection that are implicated in the transmission cycle of disease to cattle. It might be logical to conclude therefore, that the improved scientific knowledge base should lead to more rational and manageable control options. However, where these affected wildlife species have a high societal value, it has created a new set of stakeholders with often conflicting perspectives that is redolent of the antagonism among interested parties in the early twentieth century. Zoonotic TB may no longer be the potent driver for disease control that it was in previous decades. Instead, the rationale for TB eradication is now driven mainly by economic, trade, animal rights and conservation concerns (18, 132, 160). Each of these drivers brings elements of different moral frameworks and ethical perspectives, which sometimes clash because of the difficulties and uncertainties associated with control of the disease.

The examples of TB management in the different countries portray a range of single and multi-host wild animal systems implicated in the transmission cycle of TB that involves livestock. In most cases epidemiological investigations have helped reveal the reservoir status (maintenance or spillover) of many of the species involved, and this has informed the type of control measure applied (14, 161). How policy makers decide on the appropriate intervention strategies to address each concern is extremely difficult, but it must, by necessity, take account of the stakeholder perspectives in the local environment where the disease is proving problematic to eradicate (46). In New Zealand, for example, the economic impact of TB transmission from possums to cattle has been reduced significantly in recent years. Nevertheless, there is broad acceptance for continued culling of possums given their perceived status as an environmental pest species. Although there has been disquiet about the widespread use of sodium fluoroacetate (1080) in the environment (49), studies have shown high societal value placed on the conservation co-benefits as a result of culling (48). Adopting the rationale of McCulloch and Reiss (89), there is measurable net utility benefit to New Zealand biodiversity, ecology and agriculture arising from culling of possums, which validates the utilitarian approach to solve the problem.

In contrast, the culling of badgers in the UK is not short of controversy and reflects the polarized perspectives and viewpoints of the principal stakeholders. These would include the dairy and beef cattle industries and associated beneficiaries on one hand, and conservationists, animal rights groups and environmentalists on the other (53). The broad middle ground of opinion may be influenced by arguments from either side. All would agree that eradication of TB is a desirable goal though they might disagree on where the control programme should be focused. The issue at hand is how TB control is best achieved and what strategies are likely to be most effective (162). Despite being a protected species in the Republic of Ireland and the UK, the culling of badgers in order to reduce densities as a means of minimizing transmission to cattle has been central to the wildlife disease control programmes (72). This is justified by the positive outcomes achieved in New Zealand following culling of possums (14). Nevertheless, in the UK this has not detracted from the determination of opponents of the current policy to resist expansion of culling areas and to advocate for complete cessation of culling (53). It appears that there is a broad range of complex evidential and ethical perspectives at play among the principal actors. Arising from the RBCT, there are continued debates as to whether reactive or pro-active culling is the most effective strategy (163, 164). It is argued by some that the scientific evidence is not sufficiently strong to warrant culling policies (165). Others adopt a moral framework based on animal health and welfare (i.e., the moral harm from culling wild animals is inconsistent with empathy, compassion or benevolence) concluding that it is fundamentally unethical and inhumane to indiscriminately kill a protected wild species that is an integral part of the natural countryside (166). The impact of culling badgers on other animals also comes into play: opportunistic analysis associated with the RBCT has shown that population counts of hedgehogs doubled over a 5-year period from the start of cull, demonstrating potential ecological consequences of badger culling and the direct impact it has on other animal species (167). These viewpoints reflect the different moral and ethical frameworks underpinning the diverse range of opinions. According to Cassidy, the societal values and cultural framing of the badger in the UK as being “good” or “bad” is at the root of the polarized opinions on how to deal with the TB problem (168). In her essay she traces the conflict as far back as the sixteenth century when badgers were listed in the Tudor Vermin Act among animals believed to interfere with human activity, and attracted a bounty per head killed. The notion of badgers being a positive cultural iconic wildlife species was promulgated in early twentieth century literature, particularly through the influence of stories such as “The Wind in the Willows” (169), notwithstanding the social attitudes that lead to ambivalence over cruel practices such as badger baiting, which took place widely over many decades until recently.

The current arguments for and against culling of badgers in the UK broadly align with the opposing framings of the badger and the societal values assigned to the badger by either side of the debate (53, 168). On the one hand they play a defining role in the perceptions of a healthy natural countryside, while on the other they pose a serious economic threat to the cattle industry by virtue of their TB status. The approach of McCulloch and Reiss is of relevance here in that by comparing the consequential outcomes of different control strategies e.g., culling vs. vaccination, it does allow for a measurable impact of different policies (89). They propose that policy decisions affecting sentient animals be subject to a mandatory Animal Welfare Impact Assessment (AWIA) based on the arguments that (a) sentient animals are owed moral considerations, (b) there is public concern about how policy impacts on the welfare of animals, and (c) international treaties pay full regard to animal welfare (170). The desired endpoint is an overall policy that defines the greatest level of benefit (who benefits and by how much?) while accounting for the different moral frameworks that fuel the disputes. It is of interest to note that the level of acrimony between opposing sides appears to be much greater in England compared with Wales, Northern Ireland and the Republic of Ireland. Although there are no comparable sociological studies, it has been suggested that the controversies in England reflect in some part the traditional different attitudes to the countryside between urbanized and rural societies (168). Ireland has historically been a largely agrarian society with few large urban centers (compared to UK), and this may have informed attitudes to the badgers and to their place in the countryside. This makes it relatively less problematic to generate policies with clearly identifiable beneficiaries.

Some stakeholders have questioned the cost-benefit of continued costly surveillance of TB given that milk pasteurization is highly effective at killing *M. bovis*, and the risk of infection from infected meat is negligible (160). While the case may have merit from the viewpoint of agricultural economics, it does represent a narrow perspective on public spending on an animal health issue. Engaging the opinions of other stakeholders, as we have asserted, serves to broaden the arguments for continued surveillance. Many countries have successfully eradicated TB and there is a societal benefit to having disease-free cattle. In other parts of the developing world, pasteurization of milk or meat inspection is not routine and *M. bovis* in unpasteurized milk poses a zoonotic risk to consumers (171, 172). If developed countries are not seen to lead the way in progressing toward eradication this might dis-incentivize others to follow similar pathways.

We have shown here that the level of engagement and ethical perspectives of stakeholders can change when wildlife disease management becomes part of an eradication programme (46). One of the major problems for policy makers is identifying the main beneficiaries of any programme, simply because there are so many worthy candidates. In recent years, and driven by the need to better understand the disease, there have been many studies reporting new TB diagnostic tests for a variety of high value animal species (173–182). Knowledge of the extent of the disease in these animals is the first step in addressing the problem, which may prove to be very costly. The control of animal TB needs also to be considered in the context of the OIE “One health” strategy to control zoonotic diseases (183). This will require increased cooperation and communication between an expanded range of stakeholders engaged in human and animal health, the industry sector, conservation, ecologists, educators, farmers, and interested public etc. Reaching agreement on a common and standardized value system for animals may be extremely challenging, but it could represent a first step in devising solutions for TB that are realistic and achievable.

## Author Contributions

EG and LC contributed equally to this review including critical analysis of published data and preparation of the manuscript. The opinions expressed in this paper are solely those of the authors.

### Conflict of Interest Statement

The authors declare that the research was conducted in the absence of any commercial or financial relationships that could be construed as a potential conflict of interest.
